# Effect of anesthesia assistance on the detection rate of precancerous lesions and early esophageal squamous cell cancer in esophagogastroduodenoscopy screening: A retrospective study based on propensity score matching

**DOI:** 10.3389/fmed.2023.1039979

**Published:** 2023-03-23

**Authors:** Min Liang, Chunhong Xu, Xinyan Zhang, Zongwang Zhang, Junli Cao

**Affiliations:** ^1^Jiangsu Province Key Laboratory of Anesthesiology, Xuzhou Medical University, Xuzhou, China; ^2^Jiangsu Province Key Laboratory of Anesthesia and Analgesia Application Technology, Xuzhou Medical University, Xuzhou, China; ^3^Department of Anesthesiology, Liaocheng People’s Hospital, Liaocheng, Shandong, China; ^4^Department of Astroenterology, Liaocheng People’s Hospital, Liaocheng, Shandong, China; ^5^Department of Pathology, Liaocheng People’s Hospital, Liaocheng, Shandong, China; ^6^Department of Anesthesiology, Affiliated Hospital of Xuzhou Medical University, Xuzhou, China

**Keywords:** anesthesia assistance, esophageal squamous cell cancer, detection rate, esophagogastroduodenoscopy, retrospective; propensity score matching

## Abstract

**Background:**

Esophagogastroduodenoscopy (EGD) screening is vital for the early diagnosis of esophageal squamous cell cancer (ESCC). However, improvement in the detection rate of precancerous lesions and early ESCC with anesthesia assistance (AA) has not yet been investigated. This retrospective study aimed to evaluate the effect of AA on the detection rate of precancerous lesions and early ESCC in patients undergoing EGD screening and identify risk factors affecting the detection rate.

**Methods:**

We reviewed patients’ electronic medical records who underwent EGD screening between May 2019 and August 2020. Patients were divided into two groups based on whether they received AA: those in Group A underwent EGD screening with AA, and patients in Group O underwent EGD screening without AA. Propensity score matching (PSM) was used to account for differences in baseline characteristics. Detection rates of precancerous lesions and early ESCC were compared between the two groups following PSM. Binary logistic regression was used to identify risk factors affecting the detection rate.

**Results:**

The final analysis included 21,835 patients (Group A = 13,319, Group O = 8,516) from 28,985 patients who underwent EGD screening during the study period. Following PSM, 6009 patients remained in each group for analysis. There was no significant difference in the detection rate of precancerous lesions and early ESCC between Groups A and O (1.1% vs. 0.8%, *p* > 0.05). Binary logistic regression showed that age (50–59 years, 60–69 years and 70–79 years), higher endoscopist seniority, high-definition (HD) endoscopy, narrow-band imaging (NBI), and number of endoscopic images were all independent risk factors that affected the detection rate of precancerous lesions and early ESCC.

**Conclusion:**

There was no statistically significant difference in the detection rate of precancerous lesions and early ESCC between patients who underwent EGD screening with and without AA. All independent risk factors that affected the detection rate of precancerous lesions and early ESCC included the following: age (50–59 years, 60–69 years and 70–79 years), higher endoscopist seniority, HD endoscopy, NBI, and number of endoscopic images. Endoscopists should consider all these factors as much as possible when performing EGD screening.

## Introduction

Esophageal cancer is one of the most malignant cancers globally (1). In 2018, the global incidence of esophageal cancer was ranked seventh among malignant tumors (6.3/100,000), with a mortality rate ranking sixth (5.5/100,000). New cases and deaths in China account for 53.7 and 55.7% of global cases (2), respectively. Furthermore, esophageal cancer in China is higher than that in Europe and the United States, with esophageal squamous cell cancer (ESCC) being the most common. Most patients were diagnosed with advanced esophageal cancer owing to the absence of typical clinical symptoms at the early stage, with a five-year survival rate of less than 20% (3, 4). Fortunately, the five-year survival rate of patients with early esophageal cancer can be as high as 95% after treatment (5). It has been demonstrated that esophagogastroduodenoscopy (EGD) screening for high-risk groups of esophageal cancer can effectively reduce the incidence and mortality of esophageal cancer (6, 7). Hence, improving the early diagnostic yield of precancerous lesions and early ESCC is becoming urgent for patient survival.

Early diagnosis rate of esophageal cancer has been advised to be one of the quality indicators for EGD screening. There are numerous factors affecting the quality of EGD screening, including sedation, pre-endoscopy preparation, systemic examination, examination duration, endoscopy biopsy, image-enhanced endoscopy, computer-assisted diagnosis, and artificial intelligence systems (8, 9). Although anesthesia assistance (AA) has been proven to increase the detection rate of gastric lesions (10), the evidence regarding the effect of AA on the detection rate of precancerous lesions and early ESCC in patients undergoing EGD screening is rare. Thus, we used a retrospective approach to observe whether AA affects the detection rate of precancerous lesions and early ESCC in patients undergoing EGD screening.

We hypothesized that AA would improve the detection rate of precancerous lesions and early ESCC in patients undergoing EGD screening. The primary purpose of this study was to compare the detection rate of precancerous lesions and early ESCC between the patients undergoing EGD screening with and without AA. The secondary purpose was to identify the risk factors affecting the detection rate of precancerous lesions and early ESCC.

## Materials and methods

This retrospective study was approved by the Ethics Committee of Liaocheng People’s Hospital (Ethics number: 2021127). The study followed the principles of the Declaration of Helsinki.

### Study population

All electronic records of patients undergoing EGD screening were obtained from the Digestive Endoscopy Center of Liaocheng People’s Hospital. Patients who underwent EGD screening between May 2019 and August 2020 were included in the study. The target of EGD screening in our institute is to screen patients with precancerous lesions and early cancer of ESCC. The criteria of the EGD screening for ESCC include the following: patients over 40 years old, patient with a family history of ESCC, patients from high incidence areas of esophageal cancer, patients with Inducement of esophageal cancer, patients with alarm symptoms. Exclusion criteria included the following: inpatients, patients with a history of precancerous lesions and early ESCC, patients with upper gastrointestinal bleeding during EGD screening, patients under the age of 40, patients with gastric retention during EGD screening, and patients who underwent upper gastrointestinal surgery.

### Variables and outcome measurements

The following information was obtained from the electronic records system (Medcare Digital Digestive Endoscopy Workstation, Medcare Digital Engineering Co., Ltd., Qingdao, China): age, gender, endoscopist seniority, endoscopic device version, image-enhanced endoscopy, number of endoscopic images, pathological results of the biopsy, recipient of AA, and concomitant lesions under EGD screening, such as ulcers, polyps, and xanthelasma, stromal, and Barrett’s esophageal lesions, and advanced cancer.

All patients received dyclonine hydrochloride mucilage (Yangtze river pharmaceutical group, Taizhou, China) before EGD screening to improve visualization. AA was performed by anesthesiologists using propofol or midazolam-fentanyl according to standard AA guidelines set by the institute. The anesthesiologist adjusted the dose of anesthetic medication according to the clinical situation and was not collected in this study. All included patients were divided into two groups (Group A or O) based on whether they received AA.

All EGD screenings were performed by 28 endoscopists who carried out at least 1,000 EGD examinations and had ≥1 year of experience before the initiation of this study. Based on their intensive training time, nine endoscopists were placed in the junior group, 11 in the intermediate group, and eight in the senior group. All endoscopists in the senior group were experts who underwent intensive training for ≥5 years.

All EGD screenings used Sonoscape or Olympus with six versions of EGD 550 (EG-550; Sonoscape, Shenzhen, China), 260 (XQ260, Q260, H260; Olympus, Tokyo, Japan), and 290 (H290, HQ290; Olympus, Tokyo, Japan) series. EGD HQ290 series was classified as high-definition (HD). The procedure details were recorded on an endoscopy database (Medcare Digital Digestive Endoscopy Workstation, Medcare Digital Engineering Co., Ltd., Qingdao, China).

Narrow-band imaging (NBI) is an image-enhanced endoscopy that includes both image-based and dyed-based techniques. It is a virtual chromoendoscopic technique that uses blue and green light to highlight abnormal neoplastic vasculatures and is based on different optic absorbability values of hemoglobin at certain wavelengths (11). All patients in our study underwent EGD screening, whether with or without NBI.

All biopsy pathology results were analyzed and diagnosed by two experienced pathologists. Difficult cases were confirmed after discussions between pathologists in the digestive subspecialty group. We used the following definition to interpret the findings.

Precancerous lesions in our study referred to pathological changes that were closely related to esophageal cancer. These mainly included intraepithelial neoplasia (IN), classified into low-grade IN (equivalent to mild and moderate dysplasia) and high-grade IN (equivalent to severe dysplasia).

Due to the inability to distinguish whether the invasive carcinoma of the esophagus confined to the mucosa or submucosa under EGD screening, early ESCC in our study referred to invasive carcinoma of the esophagus confined to the mucosa and submucosa, with or without regional lymph node metastases.

The primary endpoint of the study was to compare the detection rate of precancerous lesions and early ESCC between groups A and O following propensity score matching (PSM) analysis. The secondary endpoint was to identify the factors influencing the detection rate of precancerous lesions and early ESCC by using binary logistic regression analysis.

### Statistical analysis

The sample size was based on available data from patients who underwent EGD screening at the Digestive Endoscopy Center of Liaocheng People’s Hospital between May 2019 and August 2020. The sample size was not statistically calculated because the parameters required to estimate the sample size cannot be determined in advance without references, and the number of cases collected is sufficient for an exploratory study. The study results are presented as numbers (percentages) for categorical variables and mean ± standard deviations for continuous variables, as appropriate. The normality of data was evaluated using the normal quantile-quantile plot. Independent samples t-tests and chi-square tests were used to compare continuous variables and categorical variables between groups, respectively.

PSM was used to assess differences in baseline characteristics between the two groups and reduce the potential confounding effect of each variable. Based on the logistic regression analysis, the propensity score was defined as the probability of receiving AA. The matching variables included age, gender, endoscopist seniority, and endoscopic device version, all of which influence the probability of receiving AA. Using the nearest neighbor method with a caliper of 0.00002 of the logit of the propensity score, we matched patients at a ratio of 1:1. In the propensity-matched cohort, paired chi-square and paired rank-sum tests were used to compare the paired groups.

The binary logistic regression model was used to identify risk factors that affected the detection rate of precancerous lesions and early ESCC. All variables were adjusted in the binary logistic regression analysis using the enter method to assess the association between AA and the detection rate of precancerous lesions and early ESCC. Patients were categorized into the following age groups to satisfy the linear relationship between age and dependent variable (Logit P): 40–49 years, 50–59 years, 60–69 years, 70–79 years, and ≥ 80 years.

The data were analyzed using SPSS software (version 26.0; SPSS Inc., Armonk, NY, United States).

## Results

**Figure 1:**
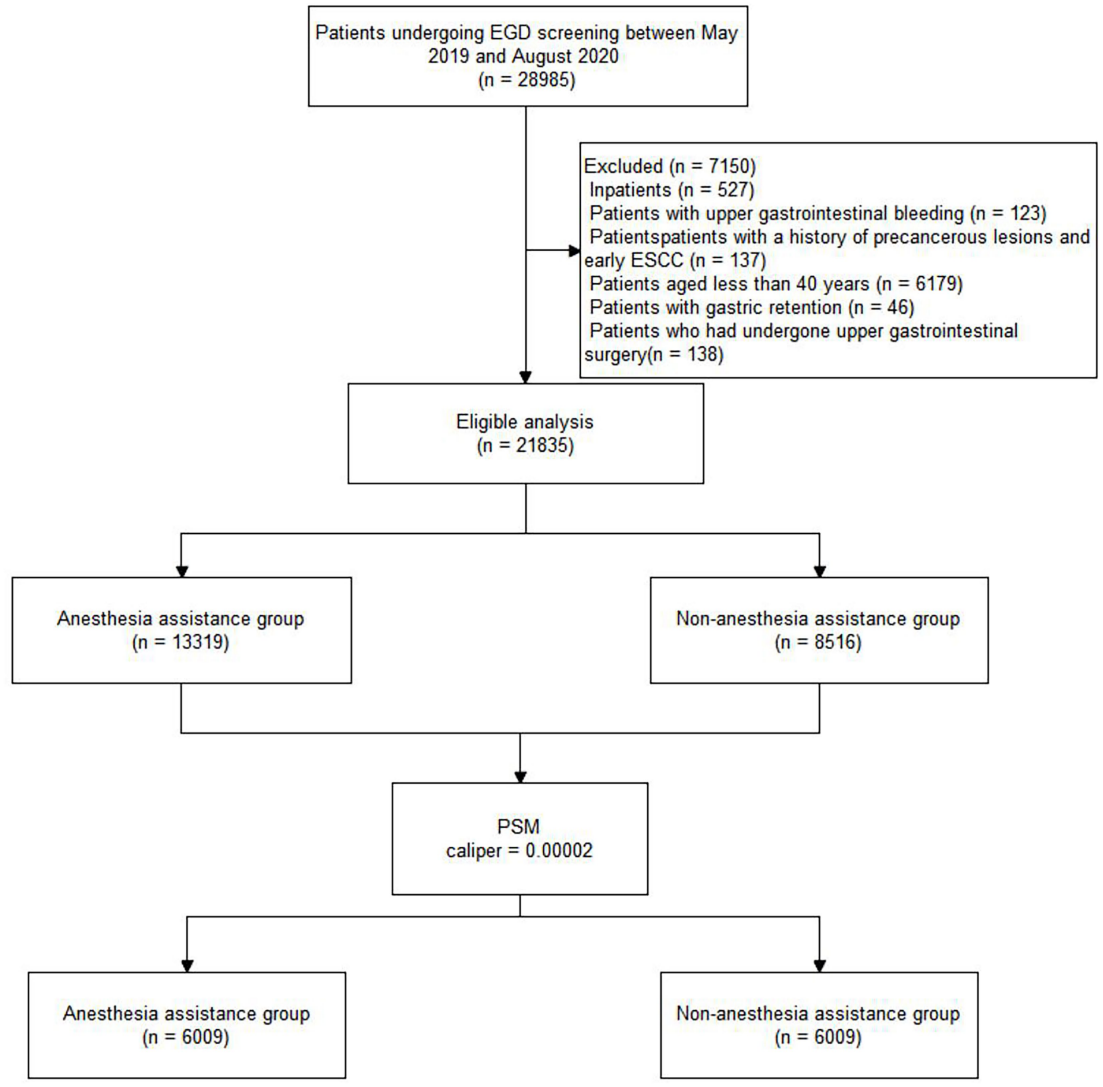
Flow diagram of the study population. EGD, esophagogastroduodenoscopy; PSM, propensity score matching.

This study was conducted at Liaocheng People’s Hospital, located in a region with a high incidence of esophageal cancer. The final analysis included 21,835 of the 28,985 patients who underwent EGD screening between May 2019 and August 2020. Patients who underwent EGD screening with AA were allocated to Group A (13,319, 61%), while patients who underwent EGD screening without AA were allocated to Group O (8,516, 39%). The distribution of patients is shown in Figure 1. Table 1 shows the characteristics of the total study cohort.

**Table 1:** Patient characteristics for the total study cohort.

Characteristic	Group A (13319)		Group O (8516)		*p-*value
	n	Proportion (%)	*n*	Proportion (%)	
Age, yr					<0.001
40–49 yr	2,943	22.1%	2,590	30.4%	
50–59 yr	5,034	37.8%	3,369	39.6%	
60–69 yr	3,633	27.3%	1921	22.6%	
70–79 yr	1,522	11.4%	592	7.0%	
≧80 yr	187	1.4%	44	0.5%	
Gender					<0.001
Male	5,758	43.2%	4,298	50.5%	
Female	7,561	56.8%	4,218	49.5%	
Endoscopist seniority					<0.001
Junior endoscopist	1,406	10.6%	3,806	44.7%	
intermediate endoscopist	9,085	68.2%	3,890	45.7%	
Senior endoscopist	2,828	21.2%	820	9.6%	
Endoscopic device version					<0.001
HD endoscopy	2,213	16.6%	472	5.5%	
Non-HD endoscopy	11,106	83.4%	8,044	94.5%	
Image enhanced endoscopy					<0.001
WLE	965	7.2%	2,366	27.8%	
NBI	12,345	92.8%	6,150	72.2%	
Number of endoscopic images	87.01 ± 26.737		81.55 ± 25.843		<0.001
Precancerous lesions and Early ESCC	272	2.0%	58	0.7%	<0.001
Barette’s esophagus	173	1.3%	129	1.5%	0.191
Advanced cancer	433	3.3%	159	1.9%	<0.001
Ucler	890	6.7%	430	5.0%	<0.001
Xanthelasma	342	2.6%	141	1.7%	<0.001
Stromal	473	3.6%	223	2.6%	<0.001
Polyp	2,398	18.0%	1,157	13.6%	<0.001

Data are presented as the median (range) or number (percentage).

PSM, propensity score matching; ESCC, esophageal squamous cell cancer; HD, high-definition; WLE, white light endoscopy; NBI, narrow-band imaging.

After PSM with a caliper of 0.00002, 6,009 patients remained in each well-matched group. The characteristics of the propensity-matched cohort are demonstrated in Table 2. In the propensity-matched cohort, there was no significant difference in the detection rate of precancerous lesions and early ESCC (1.1% vs. 0.8%, *p* > 0.05) between groups A and O (Table 2).

**Table 2:** Patient characteristics for the propensity-matched cohort.

Characteristic	Group A (6009)		Group O (6009)		*P-*value
	*n*	Proportion (%)	*n*	Proportion (%)	
Age, yr					1.000
40–49 yr	1729	28.8%	1,729	28.8%	
50–59 yr	2,384	39.7%	2,384	39.7%	
60–69 yr	1,436	23.9%	1,436	23.9%	
70–79 yr	427	7.1%	427	7.1%	
≧80 yr	33	0.5%	33	0.5%	
Gender					1.000
Male	3,097	51.5%	3,097	51.5%	
Female	2,912	48.5%	2,912	48.5%	
Endoscopist seniority					1.000
Junior endoscopist	1,302	21.7%	1,302	21.7%	
Intermediate endoscopist	3,888	64.7%	3,888	64.7%	
Senior endoscopist	819	13.6%	819	13.6%	
Endoscopic device version					1.000
Non-HD endoscopy	5,557	92.5%	5,557	92.5%	
HD endoscopy	452	7.5%	452	7.5%	
Image enhanced endoscopy					<0.001
WLE	511	8.5%	1,581	26.3%	
NBI	5,498	91.5%	4,428	73.7%	
Number of endoscopic images	79.65 ± 23.848		76.18 ± 25.253		0.011
Precancerous lesions and Early ESCC	64	1.1%	49	0.8%	0.158
Barette’s esophagus	62	1.0%	84	1.4%	0.068
Advanced cancer	174	2.9%	117	1.9%	0.001
Ucler	369	6.1%	271	4.5%	<0.001
Xanthelasma	151	2.5%	87	1.4%	<0.001
Stromal	168	2.8%	150	2.5%	0.307
Polyp	1,018	16.9%	722	12.0%	<0.001

Data are presented as the median (range) or number (percentage).

PSM, propensity score matching; ESCC, esophageal squamous cell cancer; HD, high-definition; WLE, white light endoscopy; NBI, narrow-band imaging.

In the propensity-matched cohort, univariate analyses were used to screen for variables that affected the detection rate of precancerous lesions and early ESCC. The results showed that age, gender, endoscopic device series, image-enhanced endoscopy, and number of endoscopic images were significant variables (*p* < 0.05). Subsequently, a binary logistic regression model was constructed to evaluate the association between AA and the detection rate of precancerous lesions and early ESCC (Table 3). There was no significant relationship between AA and the detection rate of precancerous lesions and early ESCC after adjusting for other factors (95% confidence interval [CI]: 0.840–1.830, *p* = 0.278).

**Table 3:** Logistic regression analysis for detection rate of precancerous lesions and early ESCC in the propensity-matched cohort.

	Detection rate, %	Unadjusted			Adjusted		
		OR	95% CI	*P*-value	OR	95% CI	*P*-value
AA							
Without AA	0.8%	Reference			Reference		
With AA	1.1%	1.309	0.901–1.903	0.157	1.240	0.840–1.830	0.278
Age							
40–49 yr	0.3%	Reference			Reference		
50–59 yr	0.7%	2.834	1.360–5.903	0.005	2.990	1.424–6.279	0.004
60–69 yr	1.7%	6.790	3.333–13.830	<0.001	6.909	3.360–14.206	<0.001
70–79 yr	2.1%	8.251	3.694–18.432	< 0.001	8.246	3.648–18.640	<0.001
≧80 yr	1.5%	5.896	0.736–47.216	0.095	4.848	0.595–39.500	0.140
Gender							
Female	0.7%	Reference			Reference		
Male	1.2%	1.808	1.232–2.652	0.002	1.393	0.937–2.071	0.102
Endoscopist seniority							
Junior endoscopist	0.5%	Reference			Reference		
Intermediate endoscopist	1.1%	1.996	1.131–3.522	0.017	2.146	1.193–3.858	0.011
Senior endoscopist	1.0%	1.825	0.888–3.749	0.102	2.311	1.096–4.872	0.028
Endoscopic device version							
Non-HD endoscopy	0.9%	Reference			Reference		
HD- endoscopy	1.7%	1.897	1.097–3.280	0.022	2.074	1.176–3.659	0.012
Image enhanced endoscopy							
WLE	0.2%	Reference			Reference		
NBI	1.1%	4.591	1.871–11.269	0.001	4.833	1.729–13.507	0.003
Number of image	/	1.021	1.016–1.026	<0.001	1.021	1.016–1.026	<0.001

Data are presented as the number (percentage).

OR, odd ratio; CI, confidence interval; AA, anesthesia assistance; ESCC, esophageal squamous cell cancer; HD, high-definition; WLE, white light endoscopy; NBI, narrow-band imaging.

Furthermore, we found that age (50–59 years, 60–69 years and 70–79 years), higher endoscopist seniority, HD endoscopy, NBI, and number of endoscopic images were all independent risk factors that affected the detection rate of precancerous lesions and early ESCC (Table 3). The detection rate of precancerous lesions and early ESCC was 2.990 times higher in patients aged 50–59 years (95% CI: 1.424–6.279, *p* = 0.004), 6.909 times higher in patients aged 60–69 years (95% CI: 3.360–14.206, *p* < 0.001), 8.246 times higher in patients aged 70–79 years (95% CI: 3.648–18.640, *p* < 0.001) compared to patients aged 40–49 years. There was no significant relationship between gender and the detection rate of precancerous lesions and early ESCC after adjusting for other factors (*p* = 0.102). Compared with junior endoscopist, the detection rate of precancerous lesions and early ESCC was 2.146 times higher by intermediate endoscopist (95% CI: 1.193–3.858, *p* = 0.011), 2.311 times higher by senior endoscopist (95% CI: 1.096–4.872, *p* = 0.028) after adjusting for other factors. The detection rate of precancerous lesions and early ESCC was 2.074 times higher using HD endoscopy (95% CI: 1.176–3.659, *p* = 0.012) compared to non-HD endoscopy. The detection rate of precancerous lesions and early ESCC was 4.833 times higher using the NBI for EGD screening (95% CI: 1.729–13.507, *p* = 0.003) compared to white light endoscopy (WLE). For every additional endoscopic image, the detection rate of precancerous lesions and early ESCC increased by 1.021-fold (95% CI: 1.016–1.026, *p* < 0.001).

## Discussion

In this study, we found no significant difference in the detection rate of precancerous lesions and early ESCC between groups A and O. After adjusting for other factors, age (50–59 years, 60–69 years and 70–79 years), higher endoscopist seniority, HD endoscopy, NBI, and the number of endoscopic images were all independent risk factors that affected the detection rate of precancerous lesions and early ESCC.

In most countries worldwide, EGD screening is one of the most important methods to screen for precancerous lesions and early ESCC. Despite widespread use, false-negative rates of EGD screenings are estimated to range between 10 and 20% (12–14), owing to the lack of guidelines and quality control (15). As a result, there is an urgent need to improve the quality of EGD screening. One of the factors influencing the quality of endoscopy examination is sedation. Several studies on colonoscopy have suggested that sedation improves adenoma detection (16), but the evidence is inconsistent (17–21). Few studies have focused on the association between AA and the rate of lesion detection in patients undergoing EGD screening. Hyun Jik Lee found that sedation does not influence the early gastric cancer detection rate (22). It is consistent with our finding. However, a retrospective study showed that EGD examination with sedation improves the detection rate of gastric polyps (3.12% vs. 5.11%, *p* < 0.05) (10). A multicenter prospective study showed that sedation with propofol improved detection rate of superficial neoplasms in patients who had undergone EGD screening (88.89% vs. 33.33%, *p* = 0.048) (23). Another multicenter study found that sedation improved the detection rate of early cancer and precancerous lesions in EGD screening (24). Hao Wu also found a higher detection rate of small upper gastrointestinal neoplasms in patients undergoing sedation-assisted EGD screening (25). These findings contradict our study. There is no significant difference in the detection rate of precancerous lesions and early ESCC between groups A and O in our study. Meanwhile, binary logistic regression analysis showed no significant relationship between AA and the detection rate of precancerous lesions and early ESCC after adjusting for other factors.

The following factors may contribute to the inconsistency: 1. This study is a single-center study. It was conducted at Liaocheng People’s Hospital, located in a region with a high incidence of esophageal cancer. The high incidence rate of ESCC may reduce the difference in detection rate of precancerous lesions and early ESCC between the two groups. 2. We only obtained the information about whether the patient received AA, but failed to collect the reasons for patients to choose AA or not. Some patients choosed EGD screening without AA due to poor economic condition or insufficient medical literacy. This may lead to an increase in patients with precancerous lesions and early ESCC in Group O. 3. Unlike other studies, ours included an endoscopic device version and image enhanced endoscopy in the analysis, which may lead to different results.

Other factors affecting the detection rate of precancerous lesions and early ESCC in our study include age (50–59 years, 60–69 years and 70–79 years), higher endoscopist seniority, HD endoscopy, NBI, and the number of endoscopic images. It has been demonstrated that the 40–79 age band (82.3% for males, 84.4% for females) is susceptible for ESCC (1985–2006) (26). Furthermore, this study reported a higher incidence of ESCC in males than in females (5.9% vs. 4.91%, *p* < 0.05) (26). It contradicts our findings. In our study, age (50–59 years, 60–69 years and 70–79 years) was an independent risk factor affecting the detection rate of precancerous lesions and early ESCC. We also found no significant difference in the detection rate of precancerous lesions and early ESCC between men and women. This inconsistency may be attributed to the fact that the objective of our study was to determine various factors influencing the detection rate of precancerous lesions and early ESCC rather than advanced ESCC. Another reason may be that the two studies included different age ranges of patients. Although endoscopy is increasingly being used for upper gastrointestinal tumor screening due to its high detection rate, the technique relies heavily on the availability of endoscopic instruments and expertise for mass screening (27). It has been demonstrated that HD endoscopy has a higher diagnostic accuracy for early gastric cancer than non - HD endoscopy (28, 29). These results align with the findings of our study, i.e., the detection rate of precancerous lesions and early ESCC is 2.074 times higher in patients undergoing EGD screening with HD endoscopy than in those having EGD screening with non-HD endoscopy. Another factor that improves the diagnostic yield of EGD screening is NBI (30). Manabu Muto found that NBI endoscopy is more sensitive and accurate than WLE in diagnosing superficial squamous cell carcinoma in the head and neck region and upper gastrointestinal tract (31). NBI is more effective and reliable for early cancer screening with EGD examination than WLE (32–34). It has been demonstrated that using non-magnifying-NBI for detecting lesions and subsequent iodine staining is an appropriate method for early cancer detection (32). NBI can replace iodine staining in screening for early ESCC for skilled endoscopists (35). This is consistent with our findings that NBI, rather than WLE, is an independent risk factor that affects the detection rate of precancerous lesions and early ESCC. Patients who underwent EGD screening with NBI had a 4.833 times higher detection rate than those who underwent WLE EGD screening. In addition, one study found that the sensitivity of NBI for screening high-grade mucosal neoplasia was 100% with the experienced endoscopists but was low with less experienced endoscopists (36). Tetsuro Yamazato found that Intensive training in EGD screening significantly improved the early gastric cancer detection rate (37). Qiang Zhang also reported that specific training could improve the endoscopic detection rate of early gastric cancer (38). It is consistent with our finding that higher endoscopist seniority is an independent risk factor affecting the detection rate of precancerous lesions and early ESCC. Furthermore, longer examinations result in higher endoscopic diagnosis yield (39). Endoscopists who carry out quick examinations may overlook neoplastic lesions in the upper gastrointestinal tract (40). It has been demonstrated that prolonged observation time could increase the neoplasm detection rate in EGD screening (41). Teh et al. (42) found that prolonging the examination time improves the lesion detection rate; Endoscopists with a mean examination time of 7 min or more were more likely to identify premalignant and neoplastic lesions during diagnostic EGD examinations. For our research, we were unable to obtain data regarding EGD screening time. As a result, we used the number of endoscopic images as a proxy for examination time in our study and found a 1.021-fold increase in the detection rate of precancerous lesions and early ESCC with every additional image.

Several limitations should be considered while interpreting the results of this study. First, using a retrospective database, some potentially influencing factors may be difficult to be collected. Considering that the enrolled patients are high-risk people, we did not analyze all risk factors related to ESCC, respectively, in this study. And we divided patients into two groups based on whether they received AA, but failed to collect the reasons for patients to choose AA or not. This would lead to selection bias and may affecting the external authenticity of the study. Second, we analyzed the number of endoscopic images rather than examination time, but this could be the result rather than the cause of increased detection. There were lesions found, and many images were taken to document the findings. It may affect the results. Third, AA included sedation with propofol and midazolam-fentanyl, which were not classified as separate in our study. A prospective study will be conducted to investigate the correlation between different types of AA and the detection rate of precancerous lesions and early ESCC. Fourth, only patients over 40 years old were included in this study. With the younger age of tumor onset, further studies are warranted in the future. Finally, this study is a single-center study, so our results can not be generalized to other medical centers.

## Conclusion

In summary, there is no statistically significant difference in the detection rate of precancerous lesions and early ESCC between patients undergoing EGD screening with and without AA. All independent risk factors that affected the detection rate of precancerous lesions and early ESCC included the following: age (50–59 years, 60–69 years and 70–79 years), higher endoscopist seniority, HD endoscopy, NBI, and number of endoscopic images. When performing EGD screening, endoscopists should consider all these factors as much as possible.

## Data availability statement

The raw data supporting the conclusions of this article will be made available by the authors, without undue reservation.

## Ethics statement

The studies involving human participants were reviewed and approved by Ethics Committee of Liaocheng People’s Hospital. Written informed consent for participation was not required for this study in accordance with the national legislation and the institutional requirements.

## Author contributions

ML and CX collected data and wrote the manuscript. XZ assisted in collecting literature and participated in discussions. ML reviewed the statistical analyses. ZZ and JC designed the study. JC and ZZ examined and verified the study. All authors read and approved the final manuscript.

## Conflict of interest

The authors declare that the research was conducted in the absence of any commercial or financial relationships that could be construed as a potential conflict of interest.

## Publisher’s note

All claims expressed in this article are solely those of the authors and do not necessarily represent those of their affiliated organizations, or those of the publisher, the editors and the reviewers. Any product that may be evaluated in this article, or claim that may be made by its manufacturer, is not guaranteed or endorsed by the publisher.

## Abbreviations

EGD: esophagogastroduodenoscopy
ESCC: esophageal squamous cell cancer
AA: anesthesia assistance
PSM: propensity score matching
HD: high-definition
NBI: narrow-band imaging
IN: intraepithelial neoplasia
WLE: white light endoscopy.
